# Supplementary material for: Navigating Uncertainties: How to Assess Welfare and Harm in Genetically Altered Animals Responsibly—A Practical Guideline
Source: Animals (Basel). 2020 May 15;10(5):857. doi: 10.3390/ani10050857 (PMC7278426; doi:10.3390/ani10050857)
Supplement: Supplementary file 1 [file animals-10-00857-s001.pdf]

File S1 – 3RsAGENT: Supplementary information and practical guidance

Questions are intended as stimulus and do not claim to be exhaustive. They are meant to assist with prospective project evaluation and severity assessment as well as with retrospective project evaluation according to actual observations.

Answers to questions should be categorised as factors of harm or modulating factors. Observations made during generation, breeding and maintenance of GA animals should be recorded for a continuous monitoring, assurance of the 3Rs and respectively for a retrospective project evaluation.

1. Genetic engineering

Potential adverse effects of different genetic engineering techniques should be kept in mind when defining factors for prospective severity assessment. The accuracy of techniques used for gene alteration has been significantly improved during the past years. In particular, genome editing and subsequent generation of GA animals has speeded up with further developments of the CRISPR/Cas9 technology [1–3]. Nevertheless, adverse effects can still occur and should be considered as uncertainty factors when generating a new line. Overall, the chosen method should be justified according to the scientific question, the intended genetic alteration, and the required number of animals. Since prospective evaluations are hypothesis-driven, the prospective severity and appropriateness of refinements have to be confirmed by an actual welfare assessment of the animals. However, a formulation of potential welfare implications prior to the generation will help to identify problems and hence improve the welfare of animals [4].

<table>
<tr><th>Prospective project evaluation</th><th>Retrospective project evaluation</th></tr>
<tr><td>Describe harm causing procedure or factor of harm:
• Which technique of genetic engineering is used?</td><td rowspan="2">List modulating factors of harm according to actual observations that have not been considered for prospective project evaluation:
• Are side effects present?
• Are animals born with the desired genotype?
• How many generations of crossing are/were needed to obtain the desired genotype including backcrossing to a specific genetic background?</td></tr>
<tr><td>Indicate modulating factors of harm that influence severity:
• a randomly genome engineering technique used with a higher probability of side effects?
• Is there a chance to use a more specific technique with less expected side effects?
• How efficient is the technique compared to other methods?</td></tr>
</table>

2. Sterile males

Infertile males are necessary to induce pseudopregnancy in female foster mice as a precondition to successful embryo transfer. Infertile males can be produced by surgical vasectomy or by breeding of infertile males. In case of surgery, the impact of the procedure and associated postsurgical pain are factors of harm and refinement strategies are essential. The procedure of surgical vasectomy is commonly classified as moderate severity. Here, a surgical access to the spermatic cord can be obtained by scrotal or abdominal access. Perioperative analgesia is required for both surgical options. Compared to the invasive procedure of vasectomy, breeding of sterile males is possible [5–7]. In both cases, the need for single-housing should be questioned to reduce stress for the male mice between mating cycles [8].

<table>
<tr><th>Prospective project evaluation</th><th>Retrospective project evaluation</th></tr>
<tr><td>Describe harm causing procedure or factor of harm:
Which method is used to produce sterile males?</td><td rowspan="2">List modulating factors of harm according to actual observations that have not been considered for prospective HBA:
Did animals recover well from anaesthesia?
Any signs of impaired well-being or delay in wound healing?</td></tr>
<tr><td>Indicate modulating factors of harm that influence severity:
In case of surgical vasectomy, is an adequate analgesia used for perioperative pain relief?
How experienced are the surgeons?
How are sterile males kept between „mating cycles? Is single-housing required?</td></tr>
</table>

3. Production of blastocystes (superovulation protocols and female donors)

In the process of creating a new mouse line or rederivation of a mouse line into an animal facility, superovulation of female donor mice and subsequent transfer of embryos to foster mothers are inevitable and frequently conducted techniques. However, appearance of harm has to be taken into account when planning and conducting such procedures. Induction of superovulation by hormone treatments with PMSG and HCG followed by collection of oocytes for in vitro fertilization (IVF) with spermatozoa is the common way to generate embryos. If IVF does not work for strain dependent reasons, natural mating of female and male mice and subsequent collection of pre-implantation embryos is another option. However, there might be differences in strain and age dependent stress

susceptibility that should be considered. Kolbe et al. [9] reported that adult C57BL6/N female mice mostly tolerated mating and copulation well, while prepubescent female mice tended to show defensive behaviour towards male mice. However, there were no differences in the level of stress hormones measured from faeces. Moreover, numbers of obtained blastocysts were significantly higher in juvenile compared to adult females, having a positive effect on animal numbers needed.

<table>
<tr><th>Prospective project evaluation</th><th>Retrospective project evaluation</th></tr>
<tr><td>Describe harm causing procedure or factor of harm:
Which method is used to obtain blastocyst?</td><td rowspan="2">List modulating factors of harm according to actual observations that have not been considered for prospective HBA:
Did superovulation work in the respective strain?
Was the yield of blastocysts sufficient?</td></tr>
<tr><td>Indicate modulating factors of harm that influence severity:
Which resources method and methods are used to produce blastocysts?
Are female donors needed to receive blastocysts or is cryopreserved material available?
Which superovulation protocol is used?
Which effect does superovulation have on females well-being?
Is IVF or natural mating performed and how will the mating affect females' well-being?</td></tr>
</table>

4. Embryo transfer and foster mothers

The transfer of embryos into the uterus of recipient female mice which serve as foster mothers is the standard procedure for rederivation or new import of strains to an animal facility by maintaining the hygienic status. Embryo transfer (ET) can be conducted nonsurgically or surgically. Positive impact on pregnancy and birth rates as well as implantation-related discomfort have been demonstrated for nonsurgical ET (NSET) [10]. However, in most institutions surgical ET is performed. Since this technique is always associated with pain and distress for the animal, adequate anaesthesia and analgesia are fundamental requirements. Moreover, success rates shown in the number of born animals versus number of transferred embryos differ notably dependent on the chosen mouse strain. Since repeated use of foster mothers has been shown to deliver consistent results [11], a second ET on the same mouse should be considered with regard to animal welfare aspects and the reduction of animal numbers.

Prospective project evaluation	Retrospective project evaluation
**Describe harm causing procedure or factor of harm:**<br>• *Which method is planned for embryo transfer?*	**List modulating factors of harm according to actual observations that have not been considered for prospective HBA:**<br>• *Did animals recover well from anesthesia?*<br>• *Any signs of impaired well-being or delay in wound healing?*<br>• *How efficient was embryo transfer (consider ratio of embryo resorption vs. born animals)?*
**Indicate modulating factors of harm that influence severity:**<br>• *Does the embryo transfer include surgical interventions or is non-surgical ET performed?*<br>• *Is unilateral or bilateral ET performed?*<br>• *Is an adequate analgesia planned for perioperative pain relief?*<br>• *Which strain is used as foster mothers and how are the expected success rates for embryo transfers?*<br>• *How experienced are the surgeons?*	

5. Phenotype characteristics

When evaluating potential harm of a genetically altered line, phenotypic characteristics will be the major component for harm assessment. Collection of information relevant databases, e.g. Mouse Genome Informatics (MGI), provide an overview on gene functions. If a new line is generated by cross-breeding, information on phenotypes of established GA mouse strains can be found online at the websites of The International Mouse Phenotyping Consortium (IMPC), the International Mouse Strain Resource (IMSR), Mouse Phenome Database (MPD) or the European Mouse Mutant Archive (EMMA), to name the most prominent resources. Most commercial breeders of laboratory animals also provide information on their websites about the strains they offer. This information also helps to estimate potential adverse effects when altering the gene of interest or crossbreeding two established strains. Nevertheless, in most cases a varying factor of uncertainty remains. A systematic actual welfare and severity assessment of animals born will lead to clarity and a line-specific description can be developed [12]. It is important to pay good attention on all phenotypic characteristics regardless of whether they affect organic functions or behavioural patterns. There are numerous studies reporting poor maternal behaviour with subsequent negative consequences for the offspring in genetically altered mice [13]. It is obligatory to consider such factors of harm when performing a systematic actual welfare and severity assessment especially in new lines.

In case of maintenance of established lines, phenotypes are well described and the harm assessment is based on information from previous breeding as well as on information on the phenotype found in the literature and databases. Nevertheless, if phenotypic data from established databases should form the major basis for severity assessment, a further analysis of data with respect to potential impairment of animal well-being is necessary. In this process, veterinarians and Animal Welfare Committees should be involved, but also scientists are in charge of investigating the correlation of phenotypic characteristics with the degree of burden of the animal [14].

When progressive disease phenotypes are present, duration and intensity of occurring pain, suffering, or distress are of special interest and need to be taken into account to assign a certain severity degree. Guidelines on severity classification of various phenotypes support researchers, animal welfare bodies, and authorities [15–17], and can support a case-by-case evaluation of experienced persons at a cage-side level.

**Prospective HBA**	**Retrospective HBA**
**Describe harm causing procedure or factor of harm:**<br>• *Which phenotype is expected?*	**List modulating factors of harm according to actual observations that have not been considered for prospective HBA:**<br>• *Can animals with progressive phenotypes be used earlier?*<br>• *What percentage of animals show a harmful phenotype?*<br>• *Is the phenotype present in different genotypes?*<br>• *Which severity degree would you assign for each genotype related phenotype?*
**Indicate modulating factors of harm that influence severity:**<br>• *Are effective refinement measures available and in place to reduce the severity of harmful phenotypes?*<br>• *How will monitoring of animals with progressive phenotypes be implemented?*<br>• *Does severity cumulate over the entire lifespan of the animal?*	

6. Hygienic and husbandry conditions

Hygienic and husbandry conditions are of great significance regarding the manifestation of phenotypes. Consideration of the hygienic status of an animal facility and in case of animal transfers, comparison of the original animal facility with the destination facility, helps to perform a prospective severity assessment of the expected phenotype. In particular, immunocompromised mouse lines, which are of huge interest for current research on the immune system, tend to respond to certain pathogens or opportunistic agents with health problems often in the digestive or respiratory system [18], which can be reflected in unwanted phenotypes. Whether or not immunocompromised animals should be considered as carrying a harmful phenotype per se has not been decided consistently across Europe.

However, there are some clear votes for the classification of breeding immunocompromised animals under a project license [12,19]. Moreover, the hygienic status of the facility might influence the development of progressive phenotypes in animals carrying harmful phenotypes per se, e.g. colitis [20,21].

Husbandry conditions comprising housing and care standards such as cage system, bedding, enrichment material and of course the expert knowledge and observation skills of animal caretakers also contribute to animal welfare. Thus, refinement measures on the husbandry level have a huge potential to ameliorate harmful phenotypes and should be examined thoroughly.

In case of an uncertain phenotype, identifications of possible hazards within the animal facility is the only appropriate measure to estimate factors of harm that might contribute to a harmful phenotype. Analysis of the hygienic status including an eighteen months health monitoring report according to FELASA recommendations [22] can give information on the absence or presence of potential pathogens that might affect the phenotypic expression of the know genotype.

Prospective project evaluation	Retrospective project evaluation
**Describe harm causing procedure or factor of harm:**<br>• *How do local hygienic and husbandry conditions influence phenotypic characteristics?*	**List modulating factors of harm according to actual observations that have not been considered for prospective HBA:**<br>• *Have unexpected observations on the phenotype according to hygiene and husbandry appeared?*
**Indicate modulating factors of harm that influence severity:**<br>• *Are hygienic barriers or housing conditions available that minimize suffering?*	

7. Breeding scheme and surplus animals

Since the appearance of a phenotype is related to the genotype, breeding schemes are sensible tools to reduce or even completely avoid animals with harmful phenotypes. In accordance with the research project and need of certain animal numbers, breeding strategies should be modified. For example, heterozygous breeding might reduce the appearance of unwanted harmful phenotypes present in homozygous animals and heterozygous mating with wildtype animals might even completely avoid harmful phenotypes. Such breeding strategies are only applicable if the genotype is known. In other cases, such as in syndromes, where identification of the genotype is part of the study and the effect of the genetic modifications on the phenotype is unclear, targeted variations of breeding methods might not help to minimize the amount of animals carrying a harmful phenotype and might be scientifically contraindicated. Taken together, the scientific value of the phenotype - is it an unwanted side effect, or the focus of the research project? - determines the range of possible breeding strategies. Moreover, the amount of surplus animals should be balanced against the number of animals that are of interest to the research project. Producing surplus animals without a harmful

phenotype that have to be sacrificed without a good reason cannot necessarily justify reduced production of animals carrying harmful phenotypes [23]. However, due to the nature of breeding it is not possible to calculate exact animal numbers with sufficient certainty to produce only those animals needed for the research project. In addition to available literature on genetics and breeding planning, several expert working groups have drawn up recommendations that provide sufficient options to reduce animal numbers [24,25].

<table>
<tr><th>Prospective project evaluation</th><th>Retrospective project evaluation</th></tr>
<tr><td>Describe harm causing procedure or factor of harm:
Which breeding scheme is planned to be used?</td><td rowspan="2">List modulating factors of harm according to actual observations that have not been considered for prospective HBA:
Can breeding scheme be optimized?</td></tr>
<tr><td>Indicate modulating factors of harm that influence severity:
Does the breeding scheme focus on the production of animals carrying less severe phenotypes?
Is a breeding scheme necessary that also produces animals with undesired/not useful genotypes?
How will surplus animals be used?</td></tr>
</table>

8. Genotyping and tissue sampling

Working with genetically altered animals requires reliable identification of the individual. There are various methods for animal identification that can be permanent or non-permanent, invasive or non-invasive and might at the same time generate tissue sampling for genotyping or not [26]. Regarding animal welfare aspects, it is always recommended to choose the least invasive method of tissue sampling that successfully identifies the animal. However, there are limiting factors regarding the applicability of some methods depending on the age of the animals. For example, distal phalanx removal can identify newborn animals with simultaneous tissue sampling at a stage of age when other methods are not applicable yet [26]. Repetition of sampling should in any case be avoided and only undertaken with non-invasive methods. Reliability of test results also plays a significant role. When non-invasive methods such as collection of fur for DNA isolation from hair follicles are used, the risk of cross contamination should be considered. Taken together, there is an obligation to minimize harmful procedures for identification and genotyping of GAA animals. FELASA recommendations for the refinement of methods for genotyping genetically modified rodents will assist in choosing an appropriate method taking animal welfare aspects into consideration [27].

Prospective project evaluation	Retrospective project evaluation
**Describe harm causing procedure or factor of harm:**<br>• *Which genotyping method will be used and what is the actual or lasting impact on the animal?*	**List modulating factors of harm according to actual observations that have not been considered for prospective HBA:**<br>• *Did the method of genotyping deliver reliable results?*<br>• *Were repeated tissue samples needed?*
**Indicate modulating factors of harm that influence severity:**<br>• *If an invasive method is used, does the method combine identification and tissue sampling?*	

1. Shen, B.; Zhang, W.; Zhang, J.; Zhou, J.; Wang, J.; Chen, L.; Wang, L.; Hodgkins, A.; Iyer, V.; Huang, X.; et al. Efficient genome modification by CRISPR-Cas9 nickase with minimal off-target effects. *Nat. Methods* **2014**, *11*, 399–402, doi:10.1038/nmeth.2857.

2. ISTT's 3Rs Committee. Will the Novel CRISPR/Cas9 Technology for the Generation of Genetically Modified Animals Increase the Number of Animals Used and Lead to a Shift in the Species Used? Statement of the ISTT's 3Rs Committee. Available online: http://www.montonerin.es/isttlegacy/isttblog/2016/05/ (accessed on 26 February 2019).

3. Seruggia, D.; Fernández, A.; Cantero, M.; Pelczar, P.; Montoliu, L. Functional validation of mouse tyrosinase non-coding regulatory DNA elements by CRISPR-Cas9-mediated mutagenesis. *Nucleic Acids Res.* **2015**, *43*, 4855–4867, doi:10.1093/nar/gkv375.

4. Dahl, K.; Sandøe, P.; Johnsen, P.F.; Lassen, J.; Hansen, A.K. Outline of a risk assessment : the welfare of future xeno-donor pigs. *Animal Welfare*, **2003**.

5. Garrels, W.; Wedekind, D.; Wittur, I.; Freischmidt, U.; Korthaus, D.; Rülicke, T.; Dorsch, M. Direct comparison of vasectomized males and genetically sterile Gapdhs knockout males for the induction of pseudopregnancy in mice. *Lab. Anim.* **2018**, *52*, 365–372, doi:10.1177/0023677217748282.

6. Davies, B.; Alghadban, S.; Preece, C.; Biggs, D.; Bouchareb, A. Replacement of surgical vasectomy by the use of wild-type sterile hybrids. In Proceedings of the 15th Transgenic Technology Meeting (TT2019), Kobe, Japan, 7–10 April 2019; p. 51.

7. Haueter, S.; Kawasumi, M.; Asner, I.; Brykczynska, U.; Cinelli, P.; Moisyadi, S.; Bürki, K.; Peters, A.H.F.M.; Pelczar, P. Genetic vasectomy-overexpression of Prm1-EGFP fusion protein in elongating spermatids causes dominant male sterility in mice. *Genesis* **2010**, *48*, 151–160, doi:10.1002/dvg.20598.

8. Pelczar, P.; Oller, H.; Bernstein, M. Permanent companion females improve the welfare of vasectomized males without adversely affection their plugging performance. In Proceedings of the 15th Transgenic Technology Meeting (TT2019), Kobe, Japan, 7–10 April 2019; p. 52.

9. Kolbe, T.; Sheety, S.; Walter, I.; Palme, R.; Rülicke, T. Impact of superovulation and mating on the wellbeing of juvenile and adult C57BL/6N mice. *Reprod. Fertil. Dev.* **2016**, *28*, 969–973, doi:10.1071/RD14372.

10. Bin Ali, R.; van der Ahé, F.; Braumuller, T.M.; Pritchard, C.; Krimpenfort, P.; Berns, A.; Huijbers, I.J. Improved pregnancy and birth rates with routine application of nonsurgical embryo transfer. *Transgenic Res.* **2014**, *23*, 691–695, doi:10.1007/s11248-014-9802-3.

11. Kolbe, T.; Palme, R.; Touma, C.; Rülicke, T. Repeated use of surrogate mothers for embryo transfer in the mouse. *Biol. Reprod.* **2012**, *86*, 1–6, doi:10.1095/biolreprod.111.092445.

12. European Commission Expert Working Group. Working Document on Genetically Altered Animals-Corrigendum of 24 January 2013. 2013. Available online: http://ec.europa.eu/environment/chemicals/lab_animals/pdf/corrigendum.pdf (accessed on 12 March 2020).

13. Kuroda, K.O.; Tachikawa, K.; Yoshida, S.; Tsuneoka, Y.; Numan, M. Neuromolecular basis of parental behavior in laboratory mice and rats: With special emphasis on technical issues of using mouse genetics. *Prog. Neuropsychopharmacol. Biol. Psychiatry* **2011**, *35*, 1205–1231.

14. Bert, B.; Chmielewska, J.; Hensel, A.; Grune, B.; Schönfelder, G. The animal experimentation quandary: Stuck between legislation and scientific freedom: More research and engagement by scientists is needed to help to improve animal welfare without hampering biomedical research. *EMBO Rep.* **2016**, *17*, 790–792, doi:10.15252/embr.201642354.

15. Home Office. Severity Classification of Genetically Altered Animals under the Animals (Scientific Procedures) Act 1986. Available online: https://www.gov.uk/government/uploads/system/uploads/attachment_data/file/276015/AdviceSeverityAssessmentGA.pdf (accessed on 12 March 2020).

16. Zintzsch, A.; Noe, E.; Reißmann, M.; Ullmann, K.; Krämer, S.; Jerchow, B.; Kluge, R.; Gösele, C.; Nickles, H.; Puppe, A.; et al. Guidelines on severity assessment and classification of genetically altered mouse and rat lines. *Lab. Anim.* **2017**, *51*, 573–582, doi:10.1177/0023677217718863.

17. Federal Food Safety and Veterinary Office FSVO. *Technical Information Animal Experimentation Severity Degrees 1.04*; Federal Food Safety and Veterinary Office FSVO: Berne, Switzerland, 2018.

18. Martins, G.A.; Cimmino, L.; Shapiro-Shelef, M.; Szabolcs, M.; Herron, A.; Magnusdottir, E.; Calame, K. Transcriptional repressor Blimp-1 regulates T cell homeostasis and function. *Nat. Immunol.* **2006**, *7*, 457–465, doi:10.1038/ni1320.

19. Chmielewska, J.; Bert, B.; Grune, B.; Hensel, A.; Schönfelder, G. Probleme aus der tierversuchsrechtlichen Praxis: Rechtliche Einordnung der Genotypisierungsmethoden sowie der Zucht immunmodifizierter Tiere. *Natur und Recht* **2017**, *39*, 385–392, doi:10.1007/s10357-017-3190-4.

20. Wilk, J.N.; Bilsborough, J.; Viney, J.L. The mdr1a$^{-/-}$ Mouse Model of Spontaneous Colitis: A Relevant and Appropriate Animal Model to Study Inflammatory Bowel Disease. *Immunol. Res.* **2005**, *31*, 151–160, doi:10.1385/IR:31:2:151.

21. Foltz, C.J.; Fox, J.G.; Cahill, R.; Murphy, J.C.; Yan, L.; Shames, B.; Schauer, D.B. Spontaneous inflammatory bowel disease in multiple mutant mouse lines: Association with colonization by Helicobacter hepaticus. *Helicobacter* **1998**, *3*, 69–78, doi:10.1046/j.1523-5378.1998.08006.x.

22. Mähler, M.; Berard, M.; Feinstein, R.; Gallagher, A.; Illgen-Wilcke, B.; Pritchett-Corning, K.; Raspa, M. FELASA recommendations for the health monitoring of mouse, rat, hamster, guinea pig and rabbit colonies in breeding and experimental units. *Lab. Anim.* **2014**, *48*, 178–192, doi:10.1177/0023677213516312.

23. Chmielewska, J.; Bert, B.; Grune, B.; Hensel, A.; Schönfelder, G. Der "vernünftige Grund" zur Tötung von überzähligen Tieren. Eine klassische Frage des Tierschutzrechts im Kontext der biomedizinischen Forschung. *Natur und Recht* **2015**, *37*, 677–682, doi:10.1007/s10357-015-2903-9.

24. Home Office. *Efficient Breeding of Genetically Altered Animals. Assessment Framework*; Home Office: London, United Kingdom, 2016.

25. Netherlands National Committee for the Protection of Animals Used for Scientific Purposes (NCad). *Genetically Modified Animals Killed in Stock*; NCad: The Hague, Netherlands, 2015.

26. Dahlborn, K.; Bugnon, P.; Nevalainen, T.; Raspa, M.; Verbost, P.; Spangenberg, E. Report of the Federation of European Laboratory Animal Science Associations Working Group on animal identification. *Lab. Anim.* **2013**, *47*, 2–11, doi:10.1177/002367712473290.

27. Bonaparte, D.; Cinelli, P.; Douni, E.; Hérault, Y.; Maas, M.; Pakarinen, P.; Poutanen, M.; Lafuente, M.S.; Scavizzi, F. FELASA guidelines for the refinement of methods for genotyping genetically-modified rodents: A report of the Federation of European Laboratory Animal Science Associations Working Group. *Lab. Anim.* **2013**, *47*, 134–145.

File S2 – Examples on how to use the 3RsAGENT

Example 1: Generation of a new line with expected sudden cardiac death syndrome

A new line will be generated as cardiac-specific knockout of the gene of interest. The GA mouse line is expected to develop a short-term, ventricular arrhythmia followed by cardiac arrest. Information on the phenotype derives from a previous and already established line with ubiquitous knockout of the same gene. In the established line, 25% of homozygous mice died at the age of one year. The electrocardiogram of affected animals showed that the time-span between the onset of arrhythmia and the death of the animal lasts a few seconds. Mice did not show any impairment of well-being prior to the onset of arrhythmia and were found dead in cage. Animals were monitored daily and showed normal behaviour and activity levels. Histopathology did not reveal cardiac hypertrophy or dilated cardiomyopathy. A similar phenotype is expected in the new cardiac-specific knockout mouse line.

Component	Describe harm causing procedure or factor of harm	Uncertainty factor regarding harm[1]	Modulating factors of harm that influence severity[2]	Severity classification[3]
**Genetic engineering**<br>*Which technique of genetic engineering is used?*	Use of a targeted mutation method (CRISPR/Cas9) to induce a knock-out of gene XY. Targeted mutation decreases side effects of the intended mutation.	☐ low<br>☒ medium<br>☐ high	Nevertheless, side effects cannot be excluded and they could influence the phenotype.	
**Sterile males**<br>*Which method is used to produce sterile males?*	Males are vasectomized by surgical intervention (scrotal access, 0,5 cm cut, cauterization vas deferens) Duration 5-10 min. General isoflurane anaesthesia and systemic analgesia.	☒ low<br>☐ medium<br>☐ high	Refined method compared to abdominal access. Anaesthesia: Isoflurane. Analgesia: Metamizol 200 mg/kg p.o. (via drinking water 1d prior to surgery, 3d postoperative), Carprofen 5 mg/kg s.c. 1x intraoperative. Warming pad intra- and postoperative. Analgesia decreases postoperative pain and distress.<br>Between mating cycles males are kept group-housed and can be used for multiple projects.	☐ non-harmful<br>☒ mild<br>☐ moderate<br>☐ severe
**Production of blastocystes**<br>*Which method is used to obtain blastocystes?*	Superovulation of 4 weeks old C57BL/6N females by injection of hormones (1x PMSG 2,5 – 5 I.U. i.p., 1 x HCG 5 – 7,5 I.U. i.p. 48 hours later). Mice are humanely killed by	☒ low<br>☐ medium<br>☐ high	Prebuscent females produce more blastocysts compared to adults and less animals have to be used.	☐ non-harmful<br>☒ mild<br>☐ moderate<br>☐ severe

1 See Table 1. for the assignment of an uncertainty factor
2 E.g. Refinement, for more details see *3RsAGENT: Supplementary information and practical guidance*
3 Consider modulating factors of harm. The classification "non-harmful" may apply only for single procedures but is not applicable to the legal requirement of assigning an overall severity for the project.

	an overdose of anesthetics and blastocystes are harvested.			
**Embryo transfer**<br>*Which method is planned for embryo transfer?*	Surgical unilateral embryo transfer (0,5 cm lateral cut) in pseudopregnant nurse females. Wound closure with suture and clips. Duration 5-10 min. General isoflurane anaesthesia and systemic analgesia.	☒ low<br>☐ medium<br>☐ high	Anaesthesia: Isoflourane; Analgesia: Metamizol 200 mg/kg p.o. (via drinking water 1d prior to surgery), Carprofen 5 mg/kg s.c. 1x intraoperative. Warming pad intra- and postoperative. Analgesia decreases postoperative pain and distress.	☐ non-harmful<br>☐ mild<br>☒ moderate<br>☐ severe
**Phenotypic characteristics**<br>*Which phenotype is expected?*	Sudden cardiac death syndrome caused by short-term arrythmia and followed by cardiac arrest.	☐ low<br>☒ medium<br>☐ high	Structured welfare assessment to identify animals with impairments, if possible. See detailed plan below.	☐ non-harmful<br>☒ mild<br>☐ moderate<br>☐ severe
**Hygienic and husbandry conditions**<br>*How do local hygienic and husbandry conditions influence phenotypic characteristics?*	Mice are kept in IVCs under SPF conditions according to FELASA (except Helicobacter spp., Pasteurella pneumotropica, Murine Norovirus)<br>Hygienic conditions do not influence the phenotype.	☐ low<br>☒ medium<br>☐ high	No special requirements.	☒ non-harmful<br>☐ mild<br>☐ moderate<br>☐ severe
**Breeding scheme and surplus animals**<br>*Which breeding scheme is planned to use? How will surplus animals be handled?*	F0: chimeras, F1: het x het, F2 hom x hom. After establishing the line, homozygous breeding scheme, all offspring animals can be used for analysis.	☐ low<br>☒ medium<br>☐ high	Because of the precision of the targeted mutation method, it is not necessary in all cases to establish more than one line to identify possible side effects which appear less likely compared to the use of random integration methods.<br>Old breeding animals will be used for educational purposes.	
**Genotyping and tissue sampling**<br>*Which method for tissue sampling will be used?*	Animals are marked by ear punching and tissue is used for genotyping.	☒ low<br>☐ medium<br>☐ high	A combination of identification and tissue sampling method is used and, therefore, only one procedure is necessary. After establishing the line, no genotyping is necessary according to breeding scheme hom x hom	☐ non-harmful<br>☒ mild<br>☐ moderate<br>☐ severe

Note: fields highlighted in grey colour are not applicable.

Prospective severity classification for the project (overall severity[4]) **With overall uncertainty of**

☒ mild ☐ moderate ☐ severe ☐ low ☒ medium ☐ high

**Plan for welfare assessment (see Table 2)**	Structured welfare assessment planned<br>☐ yes ☐ no<br>If yes, describe your plan.<br><br>The welfare assessment is planned according to the medium uncertainty factor of the project. The assessment will be done by a scientist of the group and animal caretakers will be informed about the expected phenotype. If animal display any clinical signs during husbandry routine, the responsible scientist has to be informed.<br>*Time-points of assessment:* litter of neonatal animals within the first 5 days after birth, at weaning, every 4 weeks afterwards. If unexpected clinical signs occur, the frequency will be adapted and veterinarian involved.<br>*Parameters of assessment:* general appearance, body weight measurement once per month, clinical signs for cardiac insufficiency, e.g. oedema, reduced general appearance, laboured breathing<br>*Endpoints:* If clinical signs of cardiac decompensation occur (e.g.), animals will be sacrificed and a necropsy will be made.<br>The retrospective evaluation of the welfare assessment will be discussed with the veterinarian. The plan for further breeding will be communicated to the responsible animal caretaker.
**Retrospective evaluation is planned**	☒ yes ☐ no

[4] The recommended prospective severity classification assigned to procedures should be based on the highest severity anticipated for any animal on the study (see European Commission. Working Document on a Severity Assessment Framework. Available online: http://ec.europa.eu/environment/chemicals/lab_animals/pdf/Endorsed_Severity_Assessment.pdf).

Example 2: Breeding a genetically altered mouse line to study breast cancer

A trangenic mouse line has been bred for several years at a research institute and the phenotype is well known and characterized. Homozygous mice of both sexes develop autochthonous mammary gland tumors and multifocal adenocarcinomas develop over the entire mammary fat pad. Primary tumors metastasize to lymph nodes and the lung with over 80% incidence in female mice. An early onset of palpable tumors in female mice is known and occurs with a mean latency of 53 days of age. Males also develop tumors with a later age of onset. Homozygous female mice show a loss of lactation ability. Animals will be humanely killed when reaching the following endpoints: 20% body weight loss (correction with tumor weight) or Body Condition Score 2, size or location of tumors interferes with the ability to move, tumor volume more than 1500 mm^3, reduced general health condition.

Animals are observed daily and a clinical examination is performed once a week. If a mammary gland tumor reaches 1000 mm^3 of volume, tumors are measured daily. Moreover, special attention is paid to tumor development in organs other than the mammary gland.

**Component**	**Describe harm causing procedure or factor of harm**	**Uncertainty factor regarding harm**[5]	**Modulating factors of harm that influence severity**[6]	**Severity classification**[7]
**Genetic engineering**<br>*Which technique of genetic engineering is used?*	not applicable for this specific case	☐ low<br>☐ medium<br>☐ high	-	
**Sterile males**<br>*Which method is used to produce sterile males?*	not applicable for this specific case	☐ low<br>☐ medium<br>☐ high	-	☐ non-harmful<br>☐ mild<br>☐ moderate<br>☐ severe
**Production of blastocystes**<br>*Which method is used to obtain blastocystes?*	not applicable for this specific case	☐ low<br>☐ medium<br>☐ high	-	☐ non-harmful<br>☐ mild<br>☐ moderate<br>☐ severe
**Embryo transfer**<br>*Which method is planned for embryo transfer?*	not applicable for this specific case	☐ low<br>☐ medium<br>☐ high	-	☐ non-harmful<br>☐ mild<br>☐ moderate<br>☐ severe
**Phenotypic characteristics**<br>*Which phenotype is expected?*	Multifocal mammary gland tumors and metastasis to lung and lymph nodes in both sexes.	☒ low<br>☐ medium<br>☐ high	Endpoints: 20% body weight loss (correction with tumor weight) or Body Condition Score 2, size or location of tumors interferes with the ability to move,	☐ non-harmful<br>☐ mild<br>☒ moderate<br>☐ severe

5 See Table 1. for the assignment of an uncertainty factor
6 E.g. Refinement, for more details see *3RsAGENT: Supplementary information and practical guidance*
7 Consider modulating factors of harm. The classification “non-harmful” may apply only for single procedures but is not applicable to the legal requirement of assigning an overall severity for the project.

	Reduction of lactation observed in nursing females 2 weeks postpartum.		tumor volume more than 1500 mm3, reduced general health condition.<br>Supporting measure: Lactating mice are fed with high-energy nutritional supplement.	
**Hygienic and husbandry conditions**<br>*How do local hygienic and husbandry conditions influence phenotypic characteristics?*	Mice are kept in IVCs under SPF conditions according to FELASA (except Helicobacter spp., Pasteurella pneumotropica, Murine Norovirus)<br>Hygienic conditions does not influence the phenotype.	☒ low<br>☐ medium<br>☐ high	No special requirements.	☒ non-harmful<br>☐ mild<br>☐ moderate<br>☐ severe
**Breeding scheme and surplus animals**<br>*Which breeding scheme is planned to use? How will surplus animals be handled?*	Tumor-free hemizygous transgenic male x noncarrier female to ensure lactation ability. 25% hemizygous and 75% noncarrier offspring.	☒ low<br>☐ medium<br>☐ high	Noncarrier can be used for further breeding (females) and as control animals for experiments.<br>Transgenic male breeders are used several times, but are humanely killed at the onset of tumor development.	
**Genotyping and tissue sampling**<br>*Which method for tissue sampling will be used?*	Animals are marked by ear punching and tissue is used for genotyping.	☒ low<br>☐ medium<br>☐ high	A combination of identification and tissue sampling method is used and, therefore, only one procedure is necessary.	☐ non-harmful<br>☒ mild<br>☐ moderate<br>☐ severe

Note: fields highlighted in grey colour are not applicable.

Prospective severity classification for the project (overall severity[8])

☐ mild ☒ moderate ☐ severe

With overall uncertainty of

☒ low ☐ medium ☐ high

**Plan for structured welfare assessment (see Table 2)**	Structured welfare assessment planned<br>☐ yes ☒ no<br>If yes, describe your plan
**Retrospective evaluation is planned**	☒ yes ☐ no

[8] The recommended prospective severity classification assigned to procedures should be based on the highest severity anticipated for any animal on the study (European Commission. Working Document on a Severity Assessment Framework. Available online: http://ec.europa.eu/environment/chemicals/lab_animals/pdf/Endorsed_Severity_Assessment.pdf).
